# A novel HCP (heparin-binding protein-C reactive protein-procalcitonin) inflammatory composite model can predict severe acute pancreatitis

**DOI:** 10.1038/s41598-023-36552-z

**Published:** 2023-06-09

**Authors:** Deshuai Kong, Zhang Lei, Zhenyong Wang, Meng Yu, Jinchao Li, Wei Chai, Xiulei Zhao

**Affiliations:** Department of Hepatobiliary and Pancreatic Surgery, Cangzhou Central Hospital, Cangzhou, Hebei China

**Keywords:** Predictive markers, Medical research, Biomarkers

## Abstract

Severe acute pancreatitis (SAP) presents with an aggressive clinical presentation and high lethality rate. Early prediction of the severity of acute pancreatitis will help physicians to further precise treatment and improve intervention. This study aims to construct a composite model that can predict SAP using inflammatory markers. 212 patients with acute pancreatitis enrolled from January 2018 to June 2020 were included in this study, basic parameters at admission and 24 h after hospitalization, and laboratory results such as inflammatory markers were collected. Pearson's test was used to analyze the correlation between heparin-binding protein (HBP), procalcitonin (PCT), and C-reactive protein (CRP). Risk factors affecting SAP were analyzed using multivariate logistic regression, inflammatory marker models were constructed, and subject operating curves were used to verify the discrimination of individual as well as inflammatory marker models and to find the optimal cut-off value based on the maximum Youden index. In the SAP group, the plasma levels of HBP, CRP, and PCT were 139.1 ± 74.8 ng/mL, 190.7 ± 106.3 mg/L and 46.3 ± 22.3 ng/mL, and 25.3 ± 16.0 ng/mL, 145.4 ± 67.9 mg/L and 27.9 ± 22.4 ng/mL in non-SAP patients, with a statistically significant difference between the two groups (*P* < 0.001), The Pearson correlation analysis showed a positive correlation between the three values of HBP, CRP, and PCT. The results of the multivariate logistic regression analysis showed that HBP (OR = 1.070 [1.044–1.098], *P* < 0.001), CRP (OR = 1.010 [1.004–1.016], *P* = 0.001), and PCT (OR = 1.030[1.007–1.053], *P* < 0.001) were risk factors for SAP, and the area under the curve of the HBP-CRP-PCT model was 0.963 (0.936–0.990). The HCP model, consisting of HBP, CRP, and PCT; is well differentiated and easy to use and can predict the risk of SAP in advance.

## Introduction

The incidence of acute pancreatitis has been increasing in recent years. Although most AP patients have mild symptoms, some still develop severe diseases and high mortality rates^1,2^. Therefore, it is essential to establish a comprehensive model that effectively predicts severe acute pancreatitis, which will help to refine the treatment of high-risk patients.

Del Giudice et al.^3^ reportedly predicted organ failure in AP patients by comparing several existing clinical scoring systems, including APACHE-II, BISAP, Glasgow, and HAPS scores. However, the results were ordinary and of limited clinical use. Therefore, new methods are needed to predict the severity of AP. In recent years, some researchers have predicted severe pancreatitis by building models or scores. Yang et al.^4^ developed a nomogram to predict severe pancreatitis in pregnancy, and the area under the curve of the model performed well in both the training and validation sets. Hong et al.^5^ then constructed a Logistic regression (LR) model using high -density lipoprotein cholesterol (HDL-C) on admission and 24-h blood urea nitrogen (BUN) and serum creatinine (Scr). They were able to stratify patients with SAP, which was relatively well differentiated. Although models have been previously developed to predict SAP patients, there is still uncertainty in the metrics, and variables from different sources may exist in one model.

Severe pancreatitis is characterized by multiple precipitating causes, rapid onset, rapid progression, and many complications^6^. Most patients (80–85%) mainly develop the mainly mild disease with a self-limiting course and a mortality rate of < 1–3%, but about 20% develop moderate or severe AP whenand the mortality rate is very high, ranging from 13 to 35%. Therefore, it is essential to predict the onset of severe acute pancreatitis (SAP) better and identify high-risk factors for developing complications. Previous studies have also mentioned that CRP, PCT, and HBP are essential in predicting SAP^7–9^. A better prediction may be achieved by combining a series of inflammatory indexes in a comprehensive assessment.

Therefore, we constructed an inflammatory model for the first time by combining three different inflammatory indicators (Heparin-binding protein- C reactive protein- procalcitonin), which we used to predict the occurrence of SAP and focus on managing high-risk patients.

## Materials and methods

### Patient selection

This study included 212 patients with acute pancreatitis who presented at Cangzhou Central Hospital between January 2018 and June 2020. The diagnosis of acute pancreatitis was based on the following: the presence of radiating abdominal pain; serum amylase and/or lipase levels that are at least three times the upper limit of normal; and significant presentation on enhanced computed tomography (CT) scan or ultrasound of the abdomen. SAP was defined as organ failure lasting more than 48 h^10,11^. The diagnosis of SAP is based on clinical manifestations, laboratory tests and imaging findings^12–14^. Strict inclusion and exclusion criteria were adopted for all patients, with inclusion criteria being (1) confirmed diagnosis of AP, (2) age between 18 and 75 years, (3) first episode and admission within 24 h. Exclusion criteria were (1) acute exacerbation of chronic pancreatitis, (2) during pregnancy or lactation, (3) patients with severe mental illness, (4) patients with malignancy, and (5) incomplete clinical information. This retrospective study was approved by the Ethics Committee of Cangzhou Central Hospitaland followed the Declaration of Helsinki. All patients signed an informed consent form. All patients underwent laboratory tests such as routine blood, urine, liver and kidney functions, electrolytes, blood, and urine amylase within 24 h of admission. All underwent imaging examinations such as abdominal ultrasound and enhanced abdominal CT. The treatment plan for acute pancreatitis was decided according to the latest guidelines and also after multidisciplinary discussions among gastroenterologists, pancreatic surgeons, and imaging physicians.

### Measurement of plasma HBP, PCT, and CRP levels

HBP, also known as cationic repressor protein, is mainly found in neutrophil secretory granules and asplenophil granules, and studies have shown that it plays a specific role in the regulation of the inflammatory response and vascular leakage; PCT is a pre-peptide of calcitonin, which is a glycoprotein in nature and has no hormonal activity. Under normal metabolism of the body, PCT is mainly secreted and produced by parafollicular cells of the thyroid gland, and its content in the serum is small; if bacterial infection or sepsis occurs in the body, liver macrophages and neuroendocrine cells can secrete PCT and increase the content of this substance in the serum; CRP is an acute temporal response protein produced by hepatocytes stimulated by cytokines secreted by giant cell activation and is an essential component of the body's intrinsic immune system. Patients were collected within 1–2 days of admission. Before sample collection, patients avoided strenuous exercise and were placed in a supine position. 2 ml of fasting venous blood was collected using EDTA-K2 anticoagulated vacuum blood collection tubes and inert separator gel pro-coagulation tubes; routine blood tests were performed within 30 min for EDTA-K2 anticoagulated blood samples, and the inert separator gel pro-coagulation tubes were centrifuged at 3000 r/min for 10 min, and the supernatant was taken for CRP, PCT and HBP tests. All blood samples were tested within 2 h at room temperature (25 °C). Routine blood tests were performed by Xisenmecan XE-2100 automatic hematology analyzer; CRP and PCT were performed by Beckman Coulter AU5800 automatic biochemical analysis system using the immunoturbidimetric method and immunofluorescence method, respectively. HBP was detected by ELISA, the detection instrument was an automatic enzyme standardization instrument (BIORAD, USA), and the reagents were enzyme-linked immunosorbent assay kits (Hangzhou Zhong Han Shengtai Biotechnology Co., Ltd. and Changchun Bode Technology & Biology Co., Ltd.), and the whole test operation was carried out in strict accordance with the steps or conditions specified in the relevant operating instructions of the kits and instruments.

### Clinical data collection

Clinical data on AP patients meeting inclusion and exclusion criteria were collected from the Cangzhou Central Hospital medical record system, (1) Basic hospitalization data: gender, age, Body Mass Index (BMI), etiology, medical history, etc. (2) Laboratory tests within 24 h of admission: leukocytes, hemoglobin, platelets, urea, serum creatinine, bilirubin, HBP, CRP, and PCT (3) Complications: organ failure, infectious necrosis, persistent organ failure (4) Past medical history: hypertension, diabetes mellitus, fatty liver, smoking, alcohol consumption.

### Data analysis

The *t* test was used for continuous variables that conformed to a normal distribution, and the Wilcoxon rank sum test was used for continuous variables that did not conform to a normal distribution. All categorical variables were tested with the Chi-square test or Fisher's exact test. Variables with *P* < 0.05 were first screened by univariate logistic regression analysis and then included in multivariate logistic regression analysis to screen out risk factors and construct a composite inflammatory index model. The optimal cut-off values for different inflammatory indices were calculated by selecting the number corresponding to the maximum Youden index according to sensitivity and specificity, and the receiver operating characteristic curve (ROC) was used to determine the differentiation of individual inflammatory indices as well as the integrated inflammatory model for SAP.

Data were analyzed using SPSS 25.0 (IBM, Armonk, New York, USA), and calculated *P* values < 0.05 (both sides) were considered statistically significant. Graphs were plotted using R language (version 4.0.5) and GraphPad Prism (version: 8.0). Sample size estimation was performed using PASS (version: 11.0) prior to the study.

### Ethics and consent to participate

This research was performed in accordance with the Declaration of Helsinki and was approved by the Ethics Committee of Cangzhou Central Hospital.

## Results

### Baseline information of severe acute pancreatitis and non-severe pancreatitis patients

**Figure 1:**
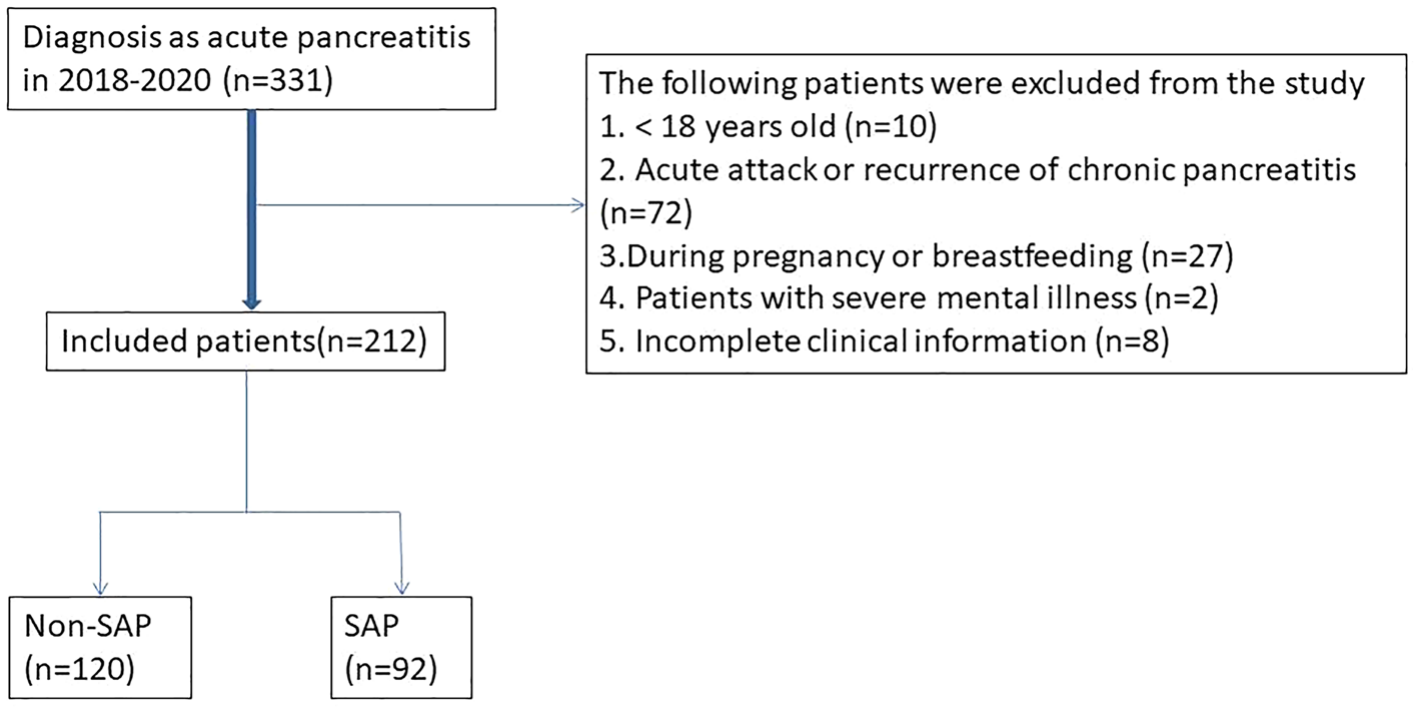
Flow chart for inclusion and exclusion of patients with acute pancreatitis.

**Figure 2:**
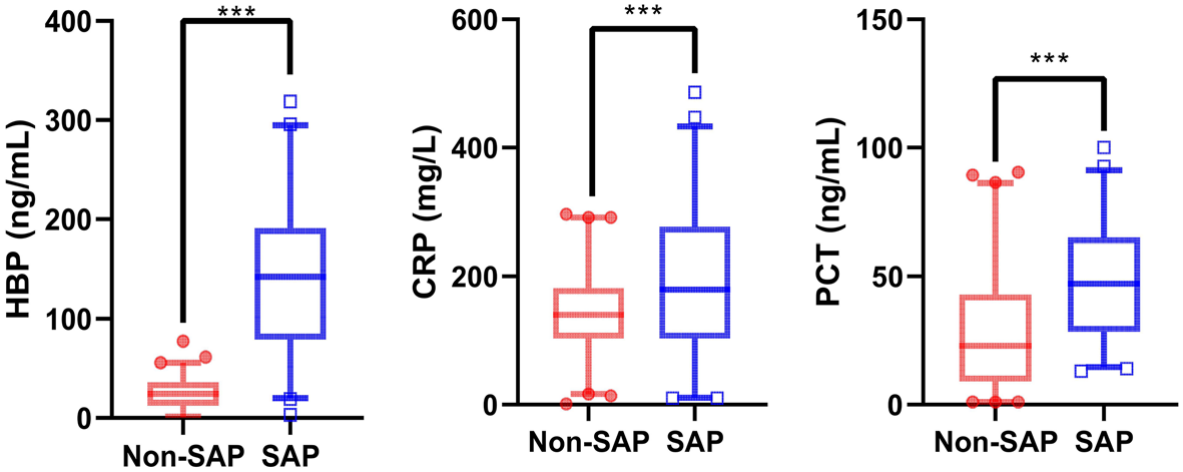
The levels of HBP, CRP, and PCT between the SAP and non-SAP groups.

A total of 212 patients were included in the study (Fig. 1), 92 in the SAP group and 120 in the Non-SAP group, with statistically significant differences in platelet count, BUN, serum creatinine, bilirubin, and international normalized ratio (INR) between the two groups in the laboratory tests (*P* < 0.001); In terms of complications, again there were statistically significant differences between the two groups, with 73 (79.3%) of the SAP patients experiencing organ failure, 55 (59.8%) experiencing infected necrosis and 70 (76.1%) experiencing persistent organ failure, all at a high rate; In termso of past medical history, 26 (28.2%) of SAP patients had diabetes and 12 (10.9%) had a history of alcohol consumption, which differed from non-SAP patients, where 8 (6.7%) had diabetes and 3 (2.5%) had a history of alcohol consumption, respectively; In terms of inflammatory indices, serum HBP, CRP and PCT values were 139.1 ± 74.8 ng/mL [142.3(79.3–191.1) ng/mL],190.7 ± 106.3 mg/L [222.3(150.0–316.6) ng/mL] and 46.3 ± 22.3 ng/mL [47.2(28.4–65.0) ng/mL] in SAP patients and 25.3 ± 16.0 ng/mL [24.4(12.4–36.0) ng/mL],145.4 ± 67.9 mg/L [132.6(88.4–175.0) ng/mL] and 27.9 ± 22.4 ng/mL [22.9(9.0–42.9) ng/mL] in non-SAP patients, respectively, all of which were statistically significantly different (*P* < 0.001) (Fig. 2). The rest of the detailed data is shown in Table 1.

**Table 1:** Demographic and clinical characteristics of patients with severe versus non-severe acute pancreatitis (n = 212).

		Non-SAP group (n = 120)	SAP group (n = 92)	*P* value^a^
Gender				0.727
	Male	98 (81.7)	73 (79.3)	
	Female	22 (18.3)	19 (20.7)	
Age				0.782
	< 50 years	62 (51.7)	50 (54.3)	
	≥ 50 years	58 (48.3)	42 (45.7)	
BMI				0.779
	< 25 kg/m^2^	72 (60.0)	53 (57.6)	
	≥ 25 kg/m^2^	48 (48.0)	39 (42.4)	
Etiology				0.001
	Biliary	39 (32.5)	40 (43.5)	
	Alcohol	28 (23.3)	12 (13.0)	
	Hypertriglyceridemia	10 (8.3)	5 (5.4)	
	Idiopathic	43 (35.8)	35 (38.0)	
Laboratory findings				
Leukocytes	× 10^9^/L	13.0 ± 5.1	13.9 ± 0.8	0.103
Hemoglobin	g/dL	10.5 ± 2.0	10.8 ± 2.5	0.333
Platelets	× 10^9^/L	156.2 ± 31.5	60.3 ± 20.8	< 0.001
BUN	mg/dL	18.1 ± 5.8	42.3 ± 11.3	< 0.001
Serum creatinine	mg/dL	0.8 ± 0.2	3.3 ± 3.5	< 0.001
Bilirubin	mg/dL	1.8 ± 1.1	8.1 ± 5.2	< 0.001
INR		1.2 ± 0.5	1.6 ± 0.6	< 0.001
Complications				
Organ failure				< 0.001
	No	112 (93.3)	19 (20.7)	
	Yes	8 (6.7)	73 (79.3)	
Infected necrosis				< 0.001
	No	93 (77.5)	37 (40.2)	
	Yes	27 (22.5)	55 (59.8)	
Persistent organ failure				< 0.001
	No	117 (97.5)	22 (23.9)	
	Yes	3 (2.5)	70 (76.1)	
Previous medical history				
Hypertension				0.243
	No	118 (98.3)	87 (94.6)	
	Yes	2 (1.7)	5 (5.4)	
Diabetes				< 0.001
	No	112 (113.3)	66 (71.8)	
	Yes	8 (6.7)	26 (28.2)	
Fatty liver disease				0.533
	No	90 (75.0)	65 (70.7)	
	Yes	30 (25.0)	27 (29.3)	
Smoking				1.000
	No	116 (96.7)	89 (96.7)	
	Yes	4 (3.3)	3 (3.3)	
Drinking				0.005
	No	117 (97.5)	80 (89.1)	
	Yes	3 (2.5)	12 (10.9)	
Inflammatory indicators				
HBP	ng/mL	25.3 ± 16.0	139.1 ± 74.8	< 0.001
CRP	mg/L	145.4 ± 67.9	190.7 ± 106.3	< 0.001
PCT	ng/mL	27.9 ± 22.4	46.3 ± 22.3	< 0.001

The values in parentheses are percentages unless indicated otherwise.

*SAP* severe acute pancreatitis, *BMI* alpha-fetoprotein, *BUN* blood urea nitrogen, *INR* international normalized ratio, *HBP* heparin-binding protein, *CRP* C-reactive protein, *PCT* procalcitonin.

^a^The χ^2^ test with Yates' correction was used for categorical data, and the *t* test was used for measures conforming to a normal distribution;

### Correlation analysis between the scores of the three inflammatory indicators

By analyzing the two-by-two Pearson coefficients between the values of the three inflammatory indices, the results showed that the Pearson coefficient was 0.374 (*P* < 0.001) between HBP and CRP, 0.327 (*P* < 0.001) between HBP and PCT, 0.212 (*P* = 001),0.002 between CRP and PCT. There was a positive correlation between the three indices (Table 2).

**Table 2:** Pearson correlation analysis.

	HBP	CRP	PCT
HBP	1		
CRP	0.374 (*P* = 0.000)	1	
PCT	0.327 (*P* = 0.000)	0.212 (*P* = 0.002)	1

*SAP* severe acute pancreatitis, *HBP* heparin-binding protein, *CRP* C-reactive protein, *PCT* procalcitonin.

### Analysis of risk factors affecting severe pancreatitis using univariate and multivariate logistic regression and construction of an inflammatory index model

The variables for which univariate logistic regression obtained *P* < 0.05 were included in the multivariate logistic regression model. Among the statistically significant variables in the univariate analysis were BMI, Leukocytes, INR, HBP, CRP, and PCT. The final multivariate logistic regression results showed that BMI > 25 (OR = 1.413, 95% CI 1.053–2.421, *P* = 0.007 vs < 25), Leukocytes (OR = 1.217, 95% CI 1.162–1.303, *P* = 0.001 per 10^9^), INR (OR = 1.244, 95% CI 1.079–1.462, *P* = 0.013 vs per 1), HBP (OR = 1.070, 95% CI 1.044–1.098, *P* < 0.001 vs per 1 ng), CRP (OR = 1.010, 95% CI 1.004–1.016, *P* = 0.001 vs per 1 mg) and PCT (OR = 1.030, 95% CI 1.007–1.053, *P* < 0.001 vs per 1 ng) were significantly associated with severe acute pancreatitis (Table 3). The HCP inflammatory index model was constructed based on the results of logistic regression with the formula = 6.850 − 0.068 × HBP (ng/mL) − 0.010 × CRP (mg/mL) − 0.029 × PCT (ng/mL).

**Table 3:** Univariate and multivariate analysis of risk factors for severe acute pancreatitis patients.

Variables		Univariate analysis			Multivariate analysis		
		*P*	OR	95% CI	*P*	OR	95% CI
Gender	Male/Female	0.112	1.151	0.913–1.355			
Age	> 50 years/≤ 50 years	0.877	1.038	0.874–1.322			
BMI	> 25/≤ 25	0.003	1.719	1.511–2.018	0.007	1.413	1.053–2.421
Leukocytes	Per 10^9^	< 0.001	1.188	1.079–1.231	0.001	1.217	1.162–1.303
Hemoglobin	Per g	0.315	0.716	0.588–1.173			
Platelets	Per 10^9^	0.371	0.973	0.833–1.138			
BUN	Per mg	0.415	1.077	0.912–1.277			
Serum creatinine	Per mg	0.078	1.002	1.000–1.013			
Bilirubin	Per mg	0.246	1.122	0.964–1.216			
INR		< 0.001	1.218	1.085–1.417	0.013	1.244	1.079–1.462
HBP	Per ng	< 0.001	1.102	1.061–1.251	< 0.001	1.070	1.044–1.098
CRP	Per mg	< 0.001	1.008	1.004–1.020	0.001	1.010	1.004–1.016
PCT	Per ng	< 0.001	1.210	1.177–1.387	< 0.001	1.030	1.007–1.053

*OR* odds ratio, *BMI* body mass index, *BUN* blood urea nitrogen, *INR* international normalized ratio, *HBP* heparin-binding protein, *CRP* C-reactive protein, *PCT* procalcitonin.

### Plotting receiver operating curves (ROC) for individual metrics as well as combined inflammatory models and the effect of each metric

**Figure 3:**
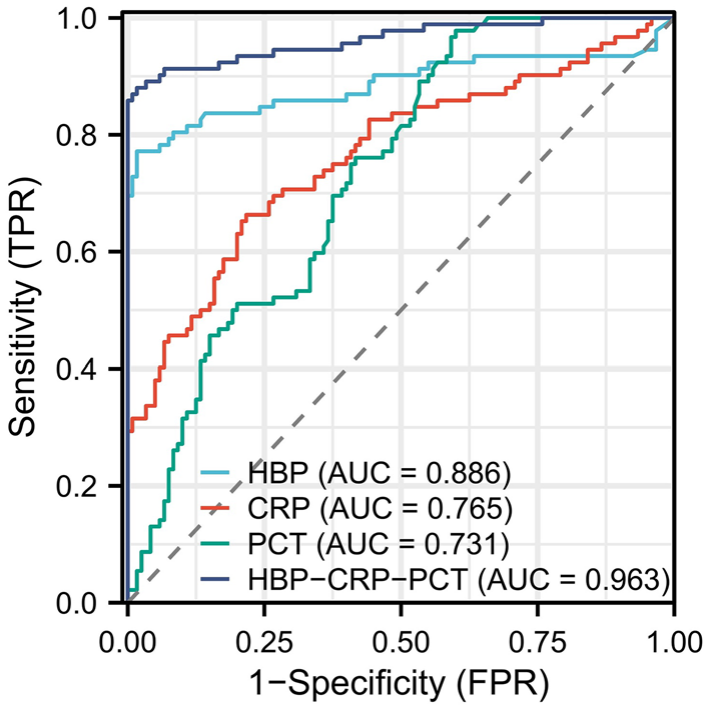
ROC curves of HBP, CRP, PCT, and combined HBP-CRP-PCT models for predicting SAP.

ROC curves were plotted for HBP, CRP, PCT, and the combined HBP-CRP-PCT model with the horizontal coordinate of 1-specificity and the vertical coordinate of sensitivity. The area under the curve was 0.886 (0.831–0.941) for HBP, 0.765 (0.699–0.832) for CRP, 0.731 (0.665–0.797) for PCT, and the area under the curve of the combined inflammatory index model was 0.963 (0.936–0.990) (Fig. 3). The optimal cut-off values for each index were determined using the maximum Youden index. The optimal cut-off values for HBP, CRP, PCT, and HBP-CRP-PCT were 56.1 ng/mL, 178.7 mg/mL, 15.6 ng/mL, and − 2.77, respectively. The sensitivity, specificity, positive predictive value, and negative predictive value were 0.772,0.983,0.973 and 0.849, respectively for HBP; 0.663,0.783,0.701 and 0.752, respectively for CRP; 0.978,0.400,0.556 and 0.960, respectively for PCT; and 0.880,0.983,0.976 and 0.915, respectively for HBP-CRP-PCT (Table 4).

**Table 4:** Predictive value of independent predictors and joint predictors for non-SAP and SAP.

Predictors	Optimal cut-off value	AUC	Accuracy	Sensitivity	Specificity	PPV	NPV
HBP	56.1	0.886 (0.831–0.941)	0.892	0.772	0.983	0.973	0.849
CRP	178.7	0.765 (0.699–0.832)	0.731	0.663	0.783	0.701	0.752
PCT	15.6	0.731 (0.665–0.797)	0.651	0.978	0.400	0.556	0.960
HBP-CRP-PCT	− 2.77	0.963 (0.936–0.990)	0.939	0.880	0.983	0.976	0.915

*SAP* severe acute pancreatitis, *HBP* heparin-binding protein, *CRP* C-reactive protein, *PCT* procalcitonin, *AUC* areas under the receiver operating characteristic curve, *PPV* positive predictive value, *NPV* negative predictive value.

### SAP patients in the 28-day death group versus the survival group and different inflammatory metrics versus models predicting 28-day death

The number of patients who died within 28 days was 37 (17.5%), and the number of patients who survived was 175 (82.5%). In the survival group, the values of HBP, CRP, and PCT were 53.4 ± 60.7, 164.9 ± 115.8 and 32.3 ± 23.4, respectively, and in the death group, the values of HBP, CRP, and PCT were 117.0 ± 85.1, 234.0 ± 147.6 and 43.1 ± 24.1, respectively, which were statistically different in both groups (Supplementary Table 1). The HBP, CRP, PCT, and HCP models predicted 28-day death in SAP patients with good discrimination, with areas under the curve of 0.728, 0.665,0.633, and 0.744, respectively (Supplementary Fig. 1).

## Discussion

In this study, a logistic model for predicting severe pancreatitis was constructed from three inflammatory indices (HBP, CRP, and PCT) in 212 patients. First, we found that all three inflammatory indices were clearly and statistically significantly correlated with the development of severe pancreatitis. Secondly, the combination of three biomarkers, HBP-CRP-PCT, could effectively distinguish severe pancreatitis from non-severe pancreatitis, and the area under the curve of the model was greater than 0.8.

At present, there are many various clinical evaluation indicators for severe pancreatitis, such as clinical manifestations, computed tomography scans, various scoring systems, and laboratory test indices^15,16^. However, there are no unified accurate diagnostic indices for severe pancreatitis. The disease of patients with severe pancreatitis progresses rapidly, and finding a test that can timely and accurately determine the patient’s condition is essential for clinical treatment and prognosis, which is also the core of the treatment of clinical acute severe pancreatitis^17–20^. It is generally accepted that inflammatory markers play an essential role in the development of severe pancreatitis. Giudice et al.^3^ suggest that CRP is an essential inflammatory indicator and affects various physiological processes. When inflammation or infection occurs, CRP concentrations rise rapidly and repair cellular tissues, reducing damage and increasing resistance to inflammation. Sager et al.^21^ evaluated several randomized controlled trials and they concluded that PCT kinetics was an indicator that was shown to correlate with the severity of pancreatitis and the degree of disease regression and that PCT had unique advantages in the management of patients. Kahn et al.^22^ investigated patients in the emergency department and they found that HBP showed good discrimination in detecting the most severe patients and also played a specific role in regulating the inflammatory response and vascular infiltration. Therefore the use of multiple inflammatory markers is necessary for the prediction of severe pancreatitis.

We constructed an inflammatory integrated logistic regression model using three inflammatory markers, heparin-binding protein, C-reactive protein, and procalcitonin. We clarified its differentiation by plotting the ROC curve, and the area under the curve of HCP was 0.963, indicating that the model can predict severe pancreatitis. Some previous studies have also combined inflammatory indicators to predict other diseases^23–25^. Niu et al.^26^ used time-resolved fluorescence immunoassay for PCT, CRP, and serum amyloid A1 and combined these three to determine early infection. The area under the curve of the combined assay was greater than 0.8. Thus this combined biomarker test could improve the diagnostic accuracy of early infection. Ma et al.^27^ used the combination of HBP and CRP to diagnose bacterial respiratory infections, and the AUC reached 0.797, also reflecting a good differentiation. This combined diagnostic score can guide the clinical formulation of a reasonable treatment plan. Yang et al.^28^ also used a triad of serum inflammatory markers to diagnose acute bacterial upper respiratory tract infections in children. This combined diagnostic score is expected to be a potential predictor of outcome. At the same time, a positive correlation was found between the expression of HBP, PCT, and CRP, which is also consistent with our study. We applied inflammatory indices to the prediction of severe pancreatitis for the first time. We found that combining multiple inflammatory indices could also predict SAP, a finding that expands the usefulness of combined inflammatory index testing.

This study has several limitations, firstly, this study is retrospective, and there is patient selection bias and confounding bias. Secondly, the data in this study are from a single center, and future studies may need to be conducted with an expanded sample size from multiple centers. Then, the established inflammatory co-detection model needs to be validated by multiple external centers to expand its applicability.

## Conclusion

In conclusion, the HCP model, which combines multiple inflammatory markers, is highly valuable for diagnosing severe acute pancreatitis and can serve as a reference for clinical management of SAP. This model aids in reducing underdiagnosis rates and enables early prediction of patient prognosis, thereby facilitating focused monitoring and treatment of high-risk patients. Overall, implementation of the HCP model can significantly improve comprehensive clinical management of SAP, enhance early diagnosis of severe acute pancreatitis, minimize delays in patient treatment, reduce adverse events, conserve medical resources, and lower patient expenses.

## Supplementary Information

Supplementary Information.

## Data Availability

The datasets used and analyzed during the current study are available from the corresponding author on reasonable request.

## References

[1] Greenberg JA (2016). Clinical practice guideline: Management of acute pancreatitis. Can. J. Surg..

[2] Lankisch PG (2015). Acute pancreatitis. Lancet (Lond. Engl.).

[3] Del Giudice M, Gangestad SW (2018). Rethinking IL-6 and CRP: Why they are more than inflammatory biomarkers, and why it matters. Brain Behav. Immun..

[4] Yang DJ (2022). Development and validation of a prediction model for moderately severe and severe acute pancreatitis in pregnancy. World J. Gastroenterol..

[5] Hong W (2017). High-density lipoprotein cholesterol, blood urea nitrogen, and serum creatinine can predict severe acute pancreatitis. Biomed. Res. Int..

[6] Leppäniemi A (2019). 2019 WSES guidelines for the management of severe acute pancreatitis. World J. Emerg. Surg. WJES.

[7] Luo Y (2021). Comprehensive mechanism, novel markers and multidisciplinary treatment of severe acute pancreatitis-associated cardiac injury— NA narrative review. J. Inflamm. Res..

[8] Staubli SM (2015). Laboratory markers predicting severity of acute pancreatitis. Crit. Rev. Clin. Lab. Sci..

[9] Zhou J (2021). Signal pathways and markers involved in acute lung injury induced by acute pancreatitis. Dis. Markers.

[10] Banks PA (2013). Classification of acute pancreatitis–2012: Revision of the Atlanta classification and definitions by international consensus. Gut.

[11] Hines OJ, Pandol SJ (2019). Management of severe acute pancreatitis. BMJ (Clin. Res. Ed).

[12] Lankisch PG (2001). Hemoconcentration: An early marker of severe and/or necrotizing pancreatitis? A critical appraisal. Am. J. Gastroenterol..

[13] Larvin M, McMahon MJ (1989). APACHE-II score for assessment and monitoring of acute pancreatitis. Lancet (Lond. Engl.).

[14] Mederos MA (2021). Acute pancreatitis: A review. JAMA.

[15] Abramczyk U (2022). Consequences of COVID-19 for the Pancreas. Int. J. Mol. Sci..

[16] Szatmary P (2022). Acute pancreatitis: Diagnosis and treatment. Drugs.

[17] Ebik B (2022). What does the procalcitonin level tell us in patients with acute pancreatitis?. J. Coll. Phys. Surg.-Pak. JCPSP.

[18] He Q (2022). The predictive value of procalcitonin combined with C-reactive protein and D dimer in moderately severe and severe acute pancreatitis. Eur. J. Gastroenterol. Hepatol..

[19] Liu P (2022). Heparin-binding protein as a biomarker of severe sepsis in the pediatric intensive care unit: A multicenter, prospective study. Clin. Chim. Acta Int. J. Clin. Chem..

[20] Xue M (2022). Clinical utility of heparin-binding protein as an acute-phase inflammatory marker in interstitial lung disease. J. Leukoc. Biol..

[21] Sager R (2017). Procalcitonin-guided diagnosis and antibiotic stewardship revisited. BMC Med..

[22] Kahn F (2019). Heparin-binding protein as a prognostic biomarker of sepsis and disease severity at the emergency department. Shock (Augusta, GA).

[23] Cai R (2021). Heparin-binding protein and procalcitonin in the diagnosis of pathogens causing community-acquired pneumonia in adult patients: A retrospective study. PeerJ.

[24] Honore PM (2021). Heparin-binding protein performs better for detecting sepsis among patients presenting with signs of systemic infection compared with procalcitonin, C-reactive protein, and lactate level, and heparin-binding protein is sufficient for both ruling in and out sepsis with organ dysfunction: beware of potential confounding factors!. Crit. Care Med..

[25] Zhou Y (2019). Usefulness of the heparin-binding protein level to diagnose sepsis and septic shock according to Sepsis-3 compared with procalcitonin and C reactive protein: A prospective cohort study in China. BMJ Open.

[26] Niu T (2019). Time-resolved fluorescent immunoassay-based combined detection of procalcitonin, C-reactive protein, heparin binding protein, and serum amyloid A1 to improve the diagnostic accuracy of early infection. J. Clin. Lab. Anal..

[27] Ma J (2022). A diagnostic test: Combined detection of heparin-binding protein, procalcitonin, and C-reactive protein to improve the diagnostic accuracy of bacterial respiratory tract infections. J. Thorac. Dis..

[28] Yang X (2022). Diagnostic value of the triple combination of serum heparin-binding protein, procalcitonin, and C-reactive protein in children with acute bacterial upper respiratory tract infection. J. Healthc. Eng..

