# Supplementary material for: A novel HCP (heparin-binding protein-C reactive protein-procalcitonin) inflammatory composite model can predict severe acute pancreatitis
Source: Sci Rep. 2023 Jun 9;13:9440. doi: 10.1038/s41598-023-36552-z (PMC10256784; doi:10.1038/s41598-023-36552-z)

# Supplementary Table 1. Comparison of inflammatory markers in the survival and dead groups within 28 days

|  | Survival | Death | P-Value |
| --- | --- | --- | --- |
| Number | 175 | 37 |  |
| HBP | 53.4±60.7 | 117.0±85.1 | <0.001 |
| CRP | 164.9±115.8 | 234.0±147.6 | 0.001 |
| PCT | 32.3±23.4 | 43.1±24.1 | 0.002 |

Abbreviation: HBP: Heparin-binding protein; CRP: C-reactive protein; PCT: procalcitonin

Supplementary Figure 1. ROC curves of HBP, CRP, PCT, and combined HBP-CRP-PCT models for predicting 28-days death.


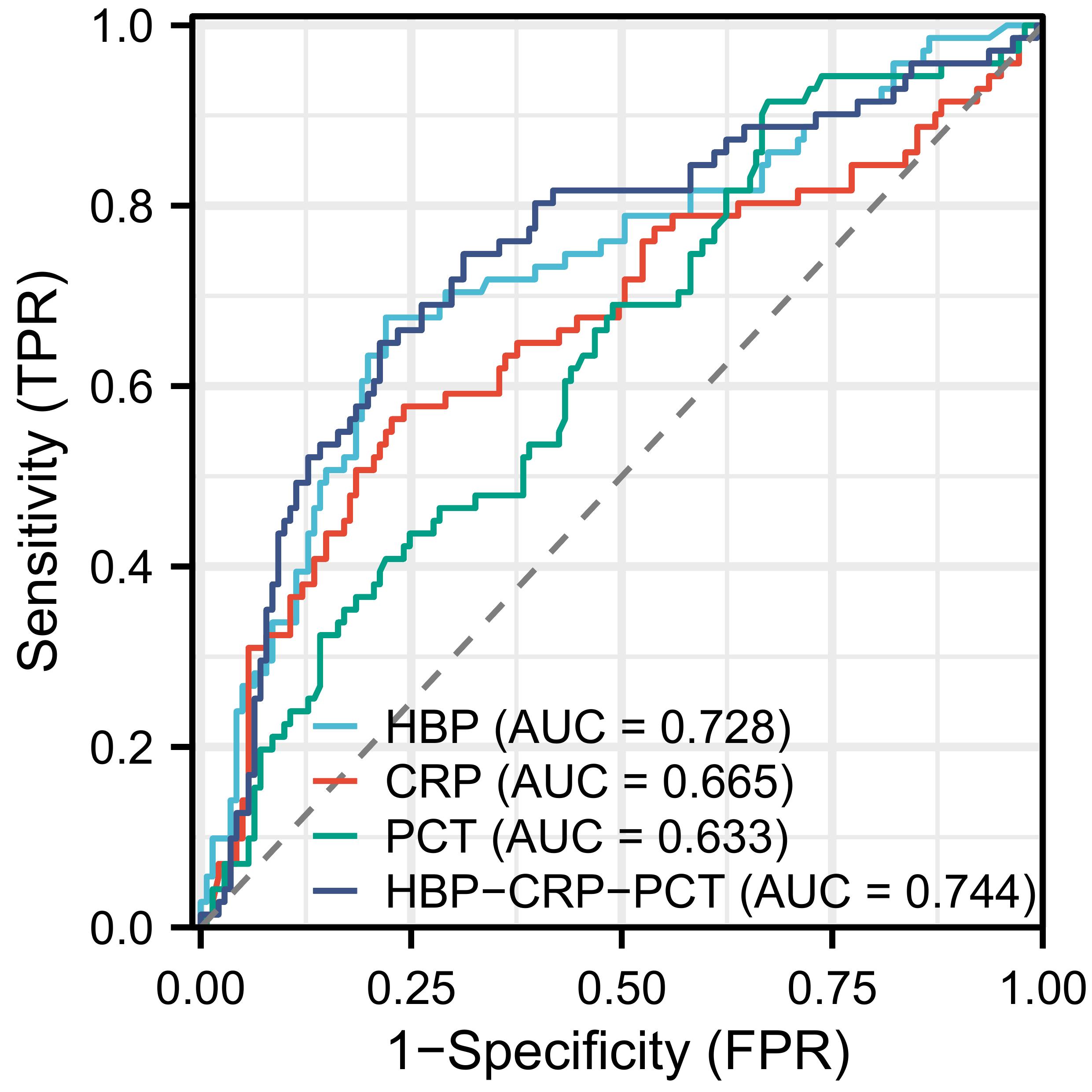

Supplement: Supplementary file 1 — Supplementary Information. [file 41598_2023_36552_MOESM1_ESM.docx]
